# Chorionic bumps in early pregnancy and their link to miscarriage

**DOI:** 10.1007/s40477-026-01209-w

**Published:** 2026-08-10

**Authors:** Melanie Norton, Jude Hamilton

**Affiliations:** Guy’s & St. Thomas’ Hospital, Westminster Bridge Rd, London, SE1 7EH UK

**Keywords:** Miscarriage, Chorionic bump, First‑trimester ultrasound, Early pregnancy, Prognostic marker

## Abstract

**Introduction:**

Chorionic bumps are a rare first‑trimester ultrasound finding with uncertain prognostic significance.

**Objective:**

To estimate the risk of miscarriage in pregnancies with a chorionic bump compared with the institutional baseline miscarriage rate in our Early Pregnancy Unit (EPU).

**Methods:**

Retrospective cohort of consecutive pregnancies with a sonographically confirmed chorionic bump (January 2020–May 2025). Outcomes were benchmarked against the EPU’s baseline miscarriage rate derived from all attendances during the study period. Prespecified secondary analyses explored whether maternal age, subchorionic haematoma (SCH), or bump size predicted outcome.

**Results:**

Among 151 pregnancies with a chorionic bump, 81 miscarried (53.6%). This was substantially higher than the EPU baseline of 14.2% (2,295 miscarriages among 16,115 patients), corresponding to a near‑sevenfold increase in odds (OR 6.99; *z* = 9.72; *p* < 0.0001). Bump size and the presence of SCH were not associated with outcome. Increasing maternal age had a modest effect (OR 1.09 per year, *p* = 0.014). Outcomes following IVF conception were similar to, or slightly better than, spontaneous conception.

**Conclusion:**

The presence of a chorionic bump is a clinically important prognostic marker of miscarriage, independent of bump size or SCH.

## Introduction

**Fig. 1:**
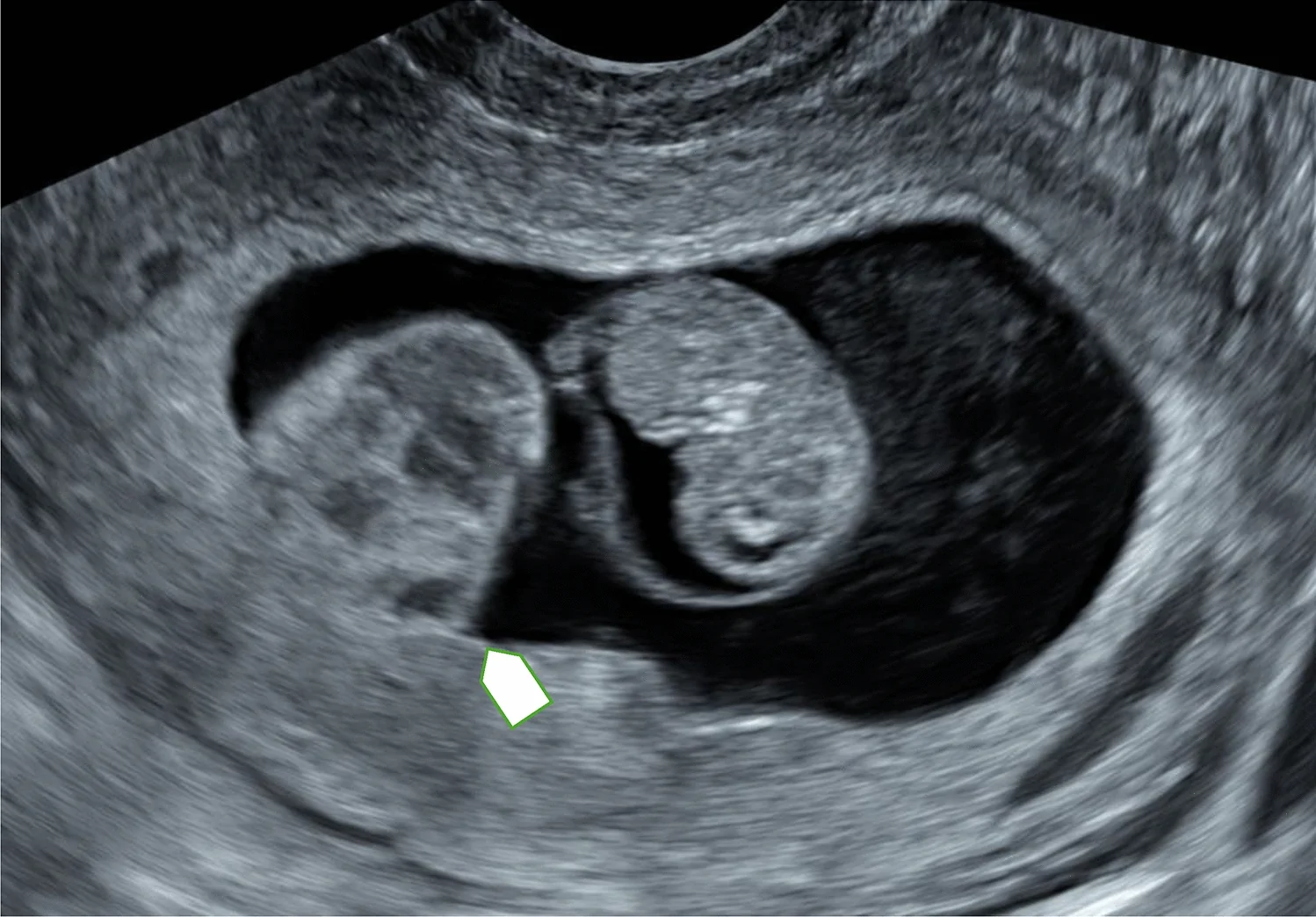
Chorionic bump (arrow) seen protruding into gestational sac next to a viable foetus on transvaginal ultrasound scan

Chorionic bumps (Fig. 1) are convex, echogenic protrusions projecting from the choriodecidual surface into the gestational sac that are usually detected (incidentally) on early transvaginal ultrasound in pregnancy. Since their first description in 2006 [1], a few studies have suggested their association with pregnancy loss [2–5]. However, the magnitude of risk and the influence of co‑findings (for example, SCH) remain uncertain [6, 7]. Clarifying prognostic significance is essential for accurate counselling in the EPU [8, 9, 10, 11].

This study evaluates outcomes for pregnancies with a chorionic bump and compares miscarriage risk to the institutional baseline derived from all EPU attendances. We also explore whether maternal age, SCH, or bump size alters risk by use of a demographic-matched control group.

## Methods

### Study design and setting

Retrospective cohort at the EPU, St Thomas’ Hospital, London, UK (January 2020–May 2025).

### Participants

#### Inclusion

Singleton pregnancies with a chorionic bump identified on first‑trimester transvaginal ultrasound.

#### Exclusion

Multiple gestation, incomplete records, uncertain outcomes.

Operational definition: a convex and echogenic protrusion into the gestational sac, clearly distinguishable from SCH.

### Data sources and variables

From anonymised records, we extracted: maternal age, BMI, ethnicity, obstetric history, conception method; gestational age at scan; ultrasound parameters (bump dimensions, CRL, Mean Sac Diamer (MSD), foetal cardiac activity, SCH); and pregnancy outcome (live birth, miscarriage, termination, molar pregnancy).

Bump dimensions were recorded in three planes (length, width, height) and averaged in millimetres. Bump locations were assessed as either above, level with, or below the yolk sac/foetal pole.

### Comparator

#### Primary comparator

The EPU baseline miscarriage rate over the study period (31,410 scans in 16,115 patients; 2,295 delayed miscarriages → 14.2%). This institutional rate was used for inferential comparisons of miscarriage risk.

#### Control cohort (descriptive only)

Women without a chorionic bump were used to compare baseline demographics/scan characteristics; their overall miscarriage rate was not used as a comparator, because it does not represent the unit’s genuine baseline risk. This comparison was performed to assess whether bump prevalence has a correlation with certain patient demographics (such as BMI, age, method of conception, etc.).

### Statistical analysis

Descriptive statistics summarised baseline characteristics and outcomes. The bump‑group miscarriage rate was compared with the 14.2% institutional baseline using a one‑sample z test for proportions; odds ratio (OR) with 95% confidence intervals (CI) was calculated. Multivariable logistic regression modelled miscarriage within the bump cohort with covariates: maternal age (per year), bump size (per mm), and SCH (present/absent). Statistical significance *p* < 0.05. Analyses in Python (v3.11) with StatsModels.

### Ethical considerations

This was a retrospective service evaluation of routinely collected, fully anonymised data; per local policy, research ethics committee approval was not required.

## Results

### Cohort and outcomes

**Fig. 2:**
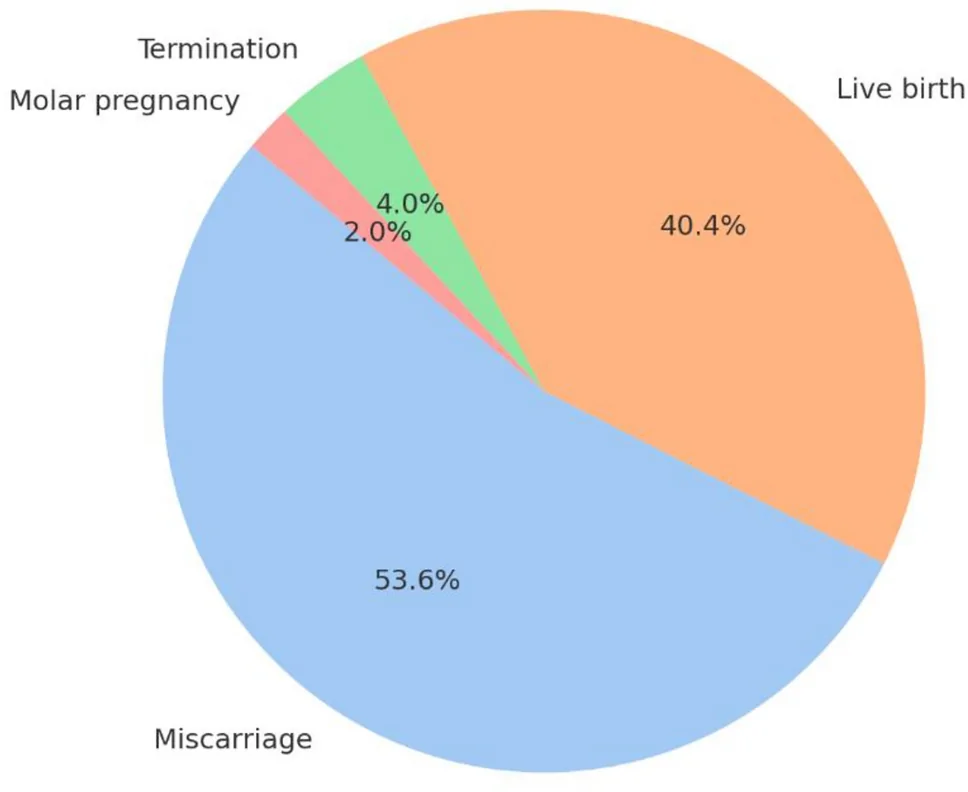
Pregnancy outcomes in chorionic bump cohort (*n* = 151)

Of 151 pregnancies diagnosed with a chorionic bump in the first trimester, outcomes were: 81 miscarriages (53.6%), 61 live births (40.4%), 6 terminations (4.0%), and 3 molar pregnancies (2.0%). Of the 81 miscarriages that occurred, 1 was a loss at 18 weeks, and 1 was a stillbirth at 36 weeks (Fig. 2).

The miscarriage rate of 53.6% (95%CI 45.7–61.4) was substantially higher than the EPU baseline of 14.2% (95%CI 13.7–14.8).

- *Absolute risk difference* + 39.4%
- *Relative risk*: 3.77 (95%CI 3.23–4.39)
- *Odds ratio (OR)* 6.99 (*z* = 9.72, *p* < 0.0001).

**Fig. 3:**
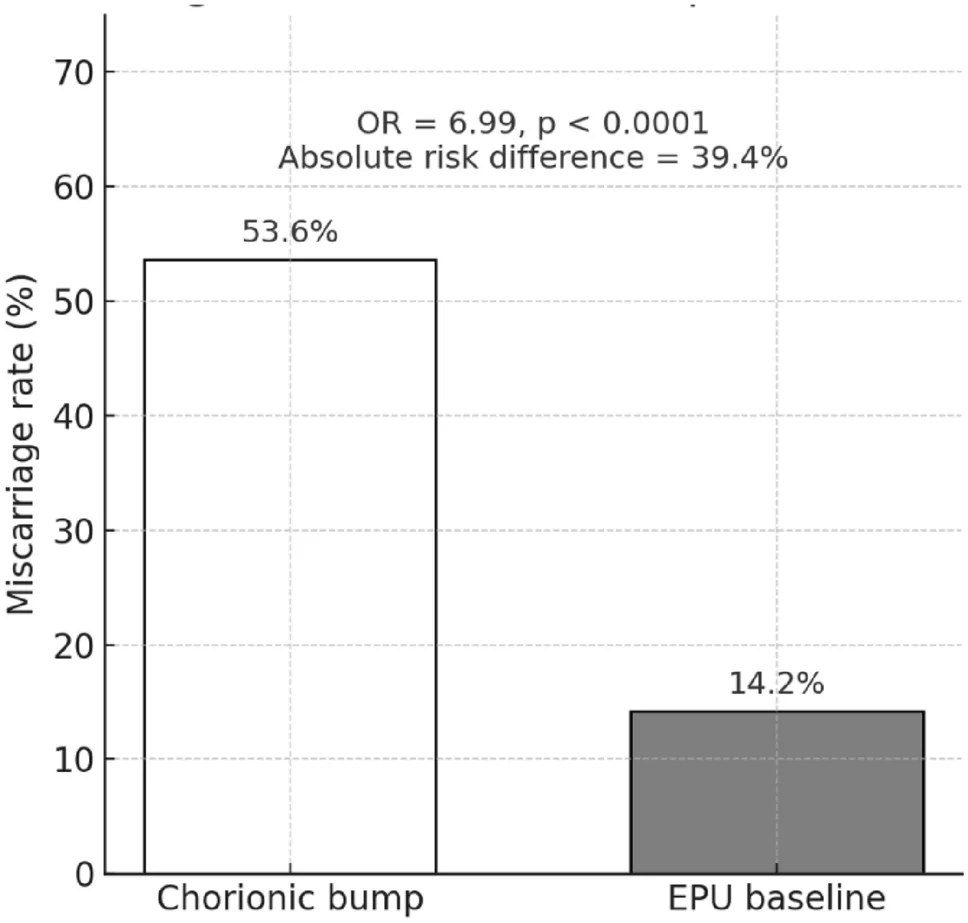
Miscarriage rate (53.6%) in bump cohort vs baseline risk (14.2%)

Thus, a chorionic bump was associated with almost a fourfold higher risk and a sevenfold higher odds of miscarriage compared with the background EPU population (Fig. 3).

### Chorionic bump characteristics and miscarriage risk

Analysis was performed to assess whether certain characteristics of a chorionic bump (such as size (mm^3^), location within the gestational sac, and combined presence of SCH) could be linked to miscarriage:

- In our cohort, chorionic bump sizes varied from 2 to 34 mm^3^ with the average bump size being 12.4 mm^3^. The chorionic bump size did not differ significantly between pregnancies that miscarried and those that did not (13.3 mm vs 11.4 mm; *p* = 0.104).
- The majority of chorionic bumps (52.1%) were found to be level with the yolk sac/foetal pole. 30.1% were found to be above and 17.8% were below the structures. Miscarriage rates were similar across bump location (Above/Level/Below; *χ*^2^ *p* = 0.64).
- The presence of SCH was not associated with miscarriage (*χ*^2^ *p* = 1.00).

**Fig. 4:**
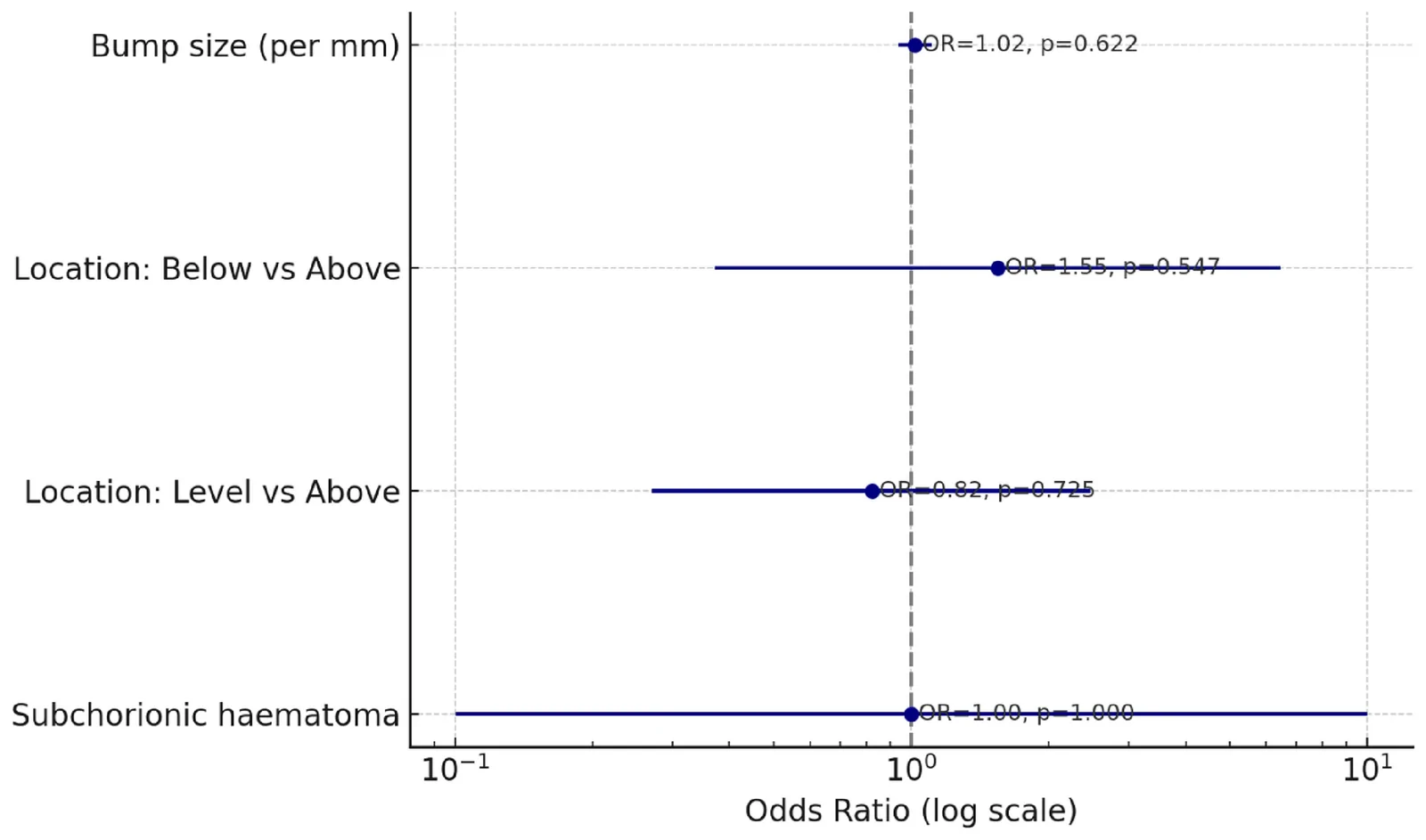
Chorionic bump features and miscarriage risk

In an exploratory multivariable model including bump size, location, and SCH, none of the variables showed a significant independent association with miscarriage Fig. 4.

### Chorionic bump occurrence in various demographics

Analysis was performed to confirm whether chorionic bumps are more common in patients with a certain demographic:

- Maternal age and BMI were similar between the chorionic bump cohort and controls (34.0 ± 5.4 vs 34.5 ± 5.1 years, *p* = 0.45; 25.5 ± 4.2 vs 26.2 ± 5.7 kg/m^2^, *p* = 0.29), suggesting that these do not increase the risk of chorionic bump occurrence.
- Rates of previous miscarriage and previous termination did not differ significantly (32.2% vs 46.3%, *p* = 0.07; 7.9% vs 10.7%, *p* = 0.67). This suggests that a patient’s history of previous miscarriage/termination does not increase the risk of chorionic bump occurrence.
- Patients who were diagnosed with a chorionic bump were significantly less likely to have had a previous live birth compared with controls (26.3% vs 40.5%, *p* = 0.048).
- Conception via IVF was equally uncommon in both groups (5.3% vs 5.0%, *p *= 1.0).
- Ethnicity distributions did not differ significantly between groups (*p* = 0.39).

**Fig. 5:**
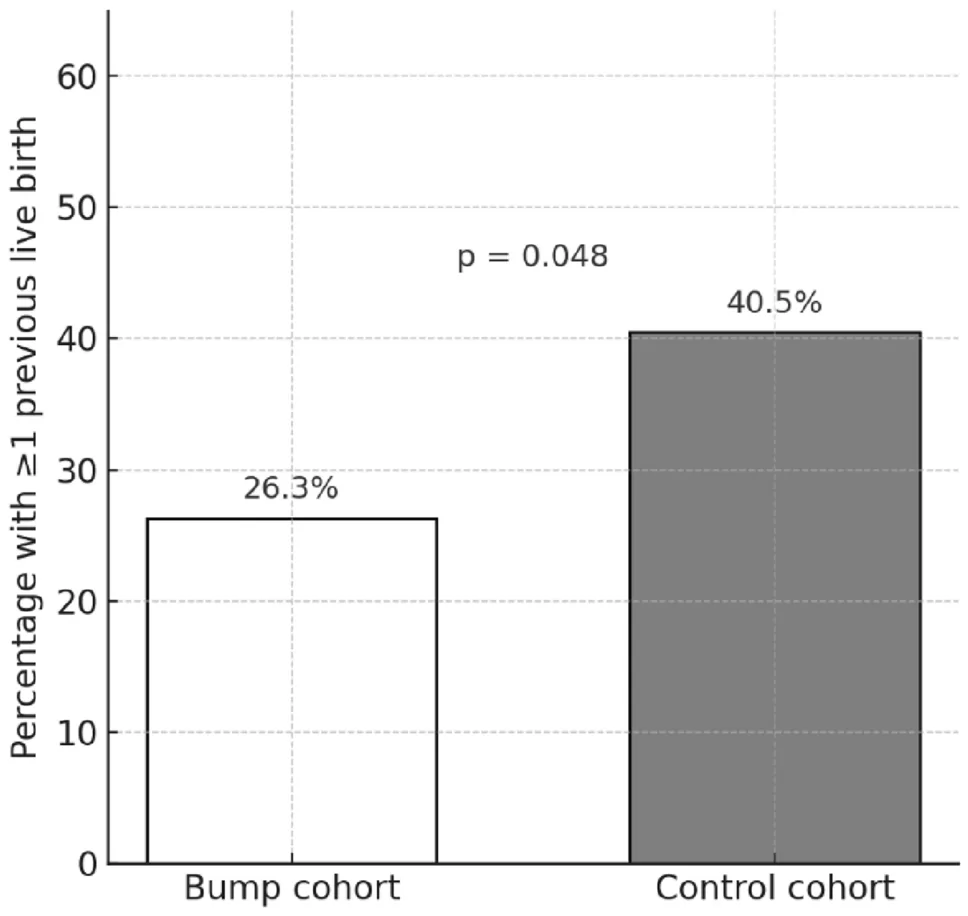
Previous live birth history: bump vs control group

In summary, the presence of a chorionic bump was not associated with maternal age, BMI, prior miscarriage, termination, IVF conception, or ethnicity, but was linked with a lower likelihood of prior live birth (Fig. 5).

## Discussion

In this 5-year cohort, chorionic bumps were strongly associated with miscarriage, with an absolute risk of 53.6% compared with a 14.2% institutional baseline [12]. This corresponded to a nearly fourfold increase in relative risk and a sevenfold increase in the odds of miscarriage. By benchmarking outcomes against a large service-wide baseline derived from more than 16,000 pregnancies assessed within our Early Pregnancy Unit, we sought to provide a clinically meaningful estimate of risk that is directly applicable to routine practice. This approach reduces many of the limitations inherent in small case–control studies, including selection bias and the use of comparator groups that may not accurately reflect the true background risk within the population being served [6, 7]. Consequently, our findings provide robust and clinically relevant data that can be used when counselling patients following the diagnosis of a chorionic bump (Table 1).

**Table 1:**
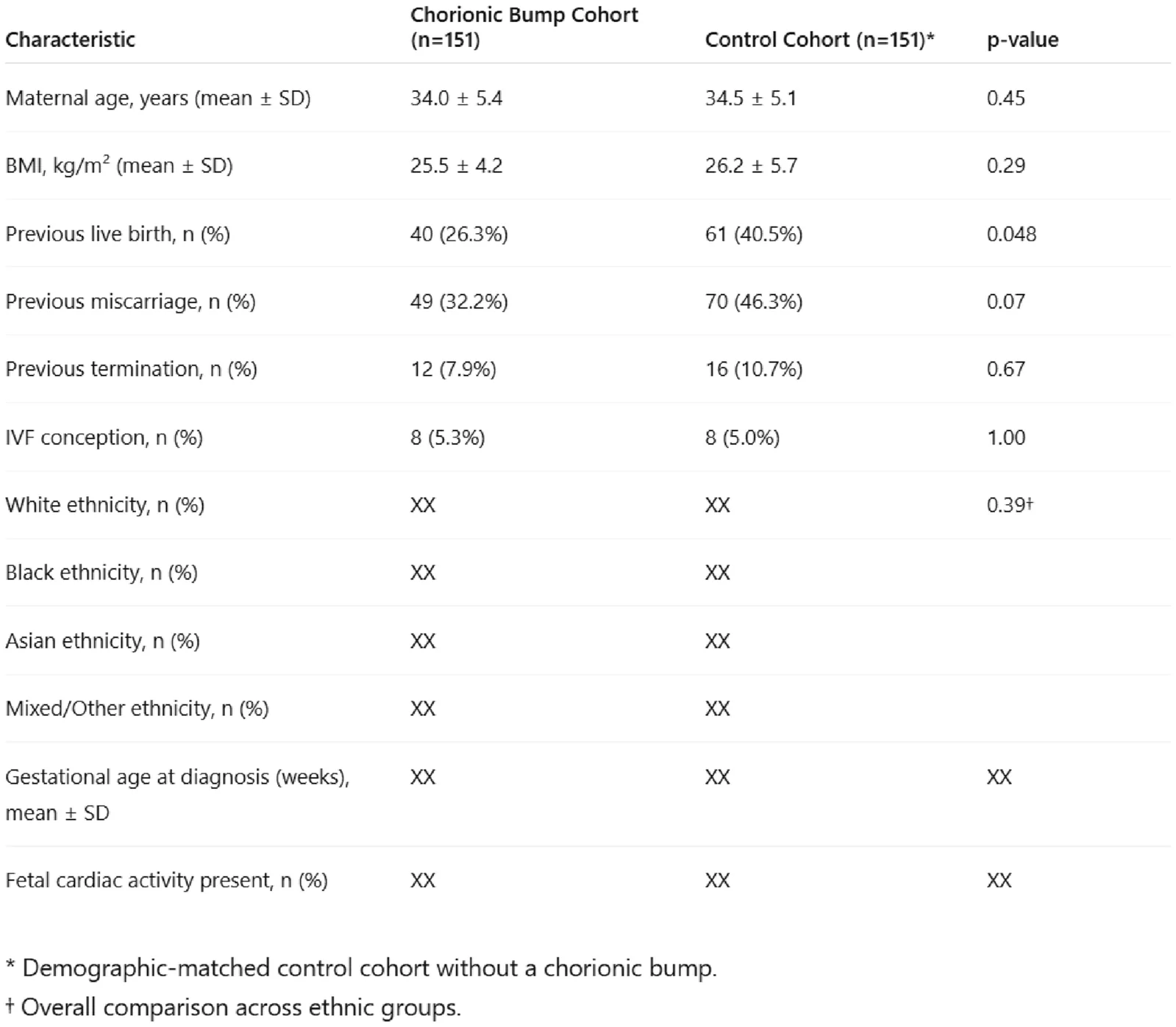
Baseline demographic and clinical characteristics of pregnancies with a chorionic bump compared with controls

Within the chorionic bump cohort, bump size, location within the gestational sac, and the presence of a subchorionic haematoma (SCH) were not associated with pregnancy outcome, reinforcing the concept that the presence of a chorionic bump itself is the principal prognostic marker rather than any specific sonographic characteristic [5, 7, 13]. Although increasing maternal age demonstrated a modest independent association with miscarriage, this finding is consistent with the well-established relationship between maternal age and pregnancy viability. Outcomes following IVF conception were not significantly worse than those observed following spontaneous conception, which is consistent with some recent reports from assisted conception populations [12, 14]. While this finding should be interpreted cautiously because of the relatively small number of IVF pregnancies included, it may reflect the increased surveillance, earlier diagnosis, and closer follow-up typically provided within fertility programmes. Interestingly, women diagnosed with a chorionic bump were significantly less likely to have had a previous live birth than controls. Although this association does not establish causality, it raises the possibility that nulliparity may be associated with an increased risk of chorionic bump formation, a finding that warrants further investigation in larger cohorts.

Our findings are consistent with extend previously published data. Since the first description of the chorionic bump by Harris et al. in 2006 [1], multiple observational studies have reported an association between chorionic bumps and adverse pregnancy outcome [2–5]. Reported miscarriage rates have generally ranged between 40 and 60%, although most published cohorts have been limited by relatively small sample sizes and, therefore, lacked statistical power to investigate factors that may modify prognosis [1–5]. A systematic review and meta-analysis by Vena et al. demonstrated an approximately threefold increase in pregnancy loss among pregnancies complicated by a chorionic bump but highlighted substantial heterogeneity between studies, including variation in study design, comparator groups, and outcome definitions [7]. More recent studies within IVF populations have similarly demonstrated increased rates of pregnancy loss, although risk estimates have varied according to patient selection and surveillance intensity [12, 14].

By analysing 151 chorionic bump pregnancies over a 5-year period, our study represents one of the larger contemporary cohorts reported in the literature [7, 12, 13]. The use of a large institutional comparator cohort allows a more precise estimation of miscarriage risk than has previously been possible and provides outcome data that are directly relevant to clinical practice. Importantly, our findings also support observations from smaller studies suggesting that sonographic characteristics such as bump size and anatomical location do not appear to influence prognosis [5, 7, 13]. This consistency across multiple cohorts strengthens the hypothesis that chorionic bumps are markers of underlying pregnancy dysfunction rather than structural abnormalities whose severity is determined by their physical dimensions.

The underlying pathophysiology of chorionic bumps remains uncertain. Histopathological reports have proposed several possible mechanisms, including focal haematoma formation, abnormal chorionic villous proliferation, localised trophoblastic degeneration, and disrupted trophoblastic invasion [5, 15]. Although the precise aetiology remains unclear, these theories share a common theme of abnormal implantation and placentation during early pregnancy. Such a mechanism is biologically plausible given the strong association observed between chorionic bumps and subsequent pregnancy loss in both our cohort and previous studies [2, 5, 7]. Furthermore, the absence of any significant relationship between prognosis and bump size, location, or coexisting SCH suggests that the bump may represent a sonographic manifestation of a broader pathological process rather than an isolated anatomical lesion. This interpretation is supported by previous authors who have proposed that chorionic bumps may be markers of impaired placental development and early placental dysfunction [5, 7]. Future prospective studies incorporating placental histopathology, first-trimester Doppler assessment, biochemical markers of placental function, and long-term obstetric outcomes may help further elucidate the biological significance of this finding.

From a clinical perspective, the identification of a chorionic bump presents a challenging counselling scenario within the Early Pregnancy Unit. While our data confirm a substantially increased risk of miscarriage, it is equally important to recognise that a considerable proportion of pregnancies remain viable, with approximately 40% progressing to live birth. This finding is consistent with the previous reports demonstrating that a chorionic bump should not be regarded as a predictor of inevitable pregnancy failure [2, 4, 7]. At present, no evidence supports any specific intervention beyond standard supportive care, and management should therefore remain consistent with routine early pregnancy practice. Nevertheless, additional ultrasound surveillance may be appropriate to provide reassurance, monitor pregnancy progression, and facilitate early identification of complications [6, 7]. The chorionic bump should, therefore, be regarded as a prognostic sonographic marker rather than a diagnosis in itself, helping clinicians provide realistic counselling while avoiding unnecessary intervention.

Strengths of this study include the use of a clearly defined ultrasound phenotype [1–3], a relatively large sample size for a rare condition [7, 12, 13], comprehensive outcome ascertainment, and comparison with a large institutional baseline derived from routine clinical practice. The inclusion of multivariable analyses allowed exploration of factors that may influence outcome while reducing the impact of confounding variables [7, 12, 16]. Furthermore, the study reflects real-world clinical practice within a high-volume tertiary Early Pregnancy Unit, enhancing the applicability of the findings to similar clinical settings.

Several limitations should be acknowledged. The retrospective design introduces the possibility of selection and information bias. As a tertiary referral centre, our population may contain a higher proportion of complex or symptomatic pregnancies than the general obstetric population, potentially limiting external validity. Although outcome data were available for the majority of patients, long-term obstetric outcomes beyond delivery were not systematically recorded. Additionally, location measurements were incomplete for a small number of cases, and detailed placental or histopathological correlation was unavailable. Finally, despite representing one of the larger cohorts reported to date [7, 12, 13], the rarity of chorionic bumps limited statistical power for certain subgroup analyses, particularly among IVF pregnancies.

## Conclusion

Chorionic bumps are a clinically important prognostic finding in early pregnancy. Affected pregnancies experienced nearly sevenfold higher odds of miscarriage compared with the EPU baseline, independent of bump size or SCH. Prospective multicentre studies should validate these findings and explore placental mechanisms.

### Key message

- Chorionic bumps are rare but prognostically important.
- Miscarriage occurred in 53.6% of bump pregnancies vs 14.2% EPU baseline (OR ≈7).
- Bump size, location, and SCH did not influence outcome.
- Bumps were less commonly seen in patients who have a previous live birth.
